# Long-term functional recovery and compensation after cerebral ischemia in rats

**DOI:** 10.1016/j.bbr.2014.05.008

**Published:** 2014-08-15

**Authors:** Sylvie Girard, Katie N. Murray, Nancy J. Rothwell, Gerlinde A.S. Metz, Stuart M. Allan

**Affiliations:** a Faculty of Life Science, University of Manchester, Manchester, UK; b Canadian Centre for Behavioural Neuroscience, Department of Neuroscience, University of Lethbridge, Lethbridge, Alberta, Canada

**Keywords:** IL-1Ra, interleukin-1 receptor antagonist, MRI, magnetic resonance imaging, MBP, myelin basic protein, SR, skilled reaching, SW, skilled walking, tMCAo, transient middle cerebral artery occlusion, Behavior, Cerebral ischemia, Skilled reaching, Fine motor analysis, IL-1Ra, Social interaction

## Abstract

Cerebral ischemia is one of the most common causes of disabilities in adults and leads to long-term motor and cognitive impairments with limited therapeutic possibilities. Treatment options have proven efficient in preclinical models of cerebral ischemia but have failed in the clinical setting. This limited translation may be due to the suitability of models used and outcomes measured as most studies have focused on the early period after injury with gross motor scales, which have limited correlation to the clinical situation. The aim of this study was to determine long-term functional outcomes after cerebral ischemia in rats, focusing on fine motor function, social and depressive behavior as clinically relevant measures. A secondary objective was to evaluate the effects of an anti-inflammatory treatment (interleukin-1 receptor antagonist (IL-1Ra)) on functional recovery and compensation. Infarct volume was correlated with long-term (25 days) impairments in fine motor skills, but not with emotional components of behavior. Motor impairments could not be detected using conventional neurological tests and only detailed analysis allowed differentiation between recovery and compensation. Acute systemic administration of IL-1Ra (at reperfusion) led to a faster and more complete recovery, but delayed (24 h) IL-1Ra treatment had no effect. In summary functional assessment after brain injury requires detailed motor tests in order to address long-term impairments and compensation processes that are mediated by intact tissues. Functional deficits in skilled movement after brain injury represent ideal predictors of long-term outcomes and should become standard measures in the assessment of preclinical animal models.

## Highlights

• Skilled reaching task allowed to differentiate between compensation and recovery.
• Infarct size correlated with long-term motor function, not with social behavior.
• Acute IL-1Ra led to recovery while compensation is seen in vehicle-treated animals.

## 1 Introduction

Acute brain injury, due to cerebral ischemia and head trauma, is one of the leading causes of death in industrialized countries and leads to long-term motor and cognitive impairments in survivors [1]. Although research has made tremendous advances in our understanding of the mechanisms leading to initial brain injury, damage progression and the implication of inflammatory processes in damage evolution, there are still no widely applicable treatments [2,3]. In part this may be due to difficulties in correlating data from pre-clinical studies with the clinical setting as a result of the heterogeneity of cerebral ischemia [4]. Although the predictive value of animal models of stroke to the human pathology is limited, recent advances in determining behavioral homologies between rat and human movements is making correlation of functional outcomes between rodents and humans feasible and more accurate [5,6].

Most preclinical studies have focused on acute outcomes after injury, mainly through the analysis of the size of the infarct at 24 h combined with basic neurological scores as a measure of acute motor impairments. Early behavioral and histological outcomes are of value although there have been controversial reports on the possible correlation between infarct size and long-term functional assessments and their predictive value for long-term outcome [7–11]. Furthermore, animals usually show rapid functional improvement after brain injury, based on their neurological score, making it more difficult to assess permanent impairments. In contrast to gross motor assessment some tests, such as the staircase test and skilled pellet reaching tasks, have proven efficient and allow detection of fine long-term impairments and can differentiate between genuine recovery and functional compensation by adopting alternate strategies [9,12–14]. The latter assessment is particularly important for a meaningful evaluation of therapeutic efficacy of treatments, and to give insight on possible roles in rehabilitation.

While motor function is of particular concern after brain injury, global patient recovery and wellness is also dependent on the presence of depression and anxiety [15–17]. The inflammatory processes that arise after brain injury are a likely cause of depression since inflammation has been shown to affect emotional behavior, including sociability, independently of the infarct itself [18–22]. Furthermore inflammatory cytokines, and particularly interleukin (IL)-1, can directly alter mood and cognition through their actions on the brain [23]. This can be transient, known as “sickness behaviour”, as well as chronic such as in case of stress-induced depression [21] or depressive disorders [24]. Since post-stroke depression affects approx. 40% of survivors and will greatly affect motor recovery and global wellbeing, it is important to include its assessment in preclinical animal models of stroke to allow comprehensive evaluation of potential therapeutics.

The objective of this study was to determine long-term functional outcomes after brain injury induced by transient middle cerebral artery occlusion (tMCAo) in rat, with special focus on fine motor skills as well as depression, sociability and anxiety. Furthermore, we assessed the long-term effects of protection against initial infarct using the anti-inflammatory interleukin-1 receptor antagonist (IL-1Ra), and whether delayed administration of IL-1Ra is also beneficial. Using a fine motor task, we showed that impairments are clearly visible 1 month after injury, despite partial infarct resolution, and that functional recovery can be separated from compensation. Ischemic injury induced both long-lasting sociability impairments and depressive-like behavior. Skilled reaching success correlated with infarct volume although there was no correlation with either sociability or depression. Systemic administration of IL-1Ra at occlusion decreased initial infarct size, enhanced recovery and provided protection against defects in sociability and depressive-like state.

## 2 Materials and methods

### 2.1 Animals

Adult male Wistar rats (obtained at 8 weeks of age, approx. 200 g; Charles River, Kent, UK) were used. All experiments were conducted under UK Animals (Scientific Procedures) Act, 1986. The animals were kept in a 12 h light–dark cycle and acclimatized for 1 week with free access to food and water prior to any manipulation.

### 2.2 Experimental design and skilled reaching training

**Fig. 1:**
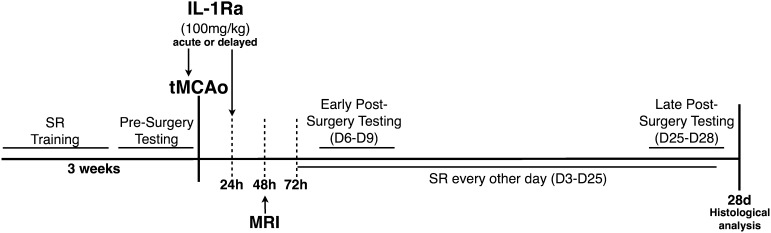
Experimental design. Animals were trained on the skilled reaching (SR) task for 3 weeks prior to brain injury induced by transient middle cerebral artery occlusion (tMCAo). Brain injury was assessed 48 h after the induction by magnetic resonance imaging (MRI). IL-1Ra was administered either acutely, at the time of occlusion, or delayed, 24 h after injury.

Animals were trained in the skilled reaching (SR) task daily for 2 weeks. To stimulate motivation, the animals were placed on a restricted diet to maintain 90% of their free feeding body weight (weight monitored twice daily). Restricted diet was used only for the first week of training after which animals were allowed free access to food and water. Four days prior to brain injury induced by tMCAo the animals were subjected to behavioral testing (as described below) in order to acquire baseline and ensure that all animals performed similarly (Fig. 1). After training animals were allowed to rest for a day prior to surgery (Fig. 1). Full behavioral testing was performed at an early time point after surgery (day (D) 6–9) and after 1 month (D25–28) (Fig. 1).

### 2.3 Focal cerebral ischemia

Brain injury was induced by 60 min tMCAo as previously described [25]. Briefly, animals (approx. 12 weeks of age) were anesthetized with isoflurane (4% induction and 1.5% maintenance in 70:30 N_2_O/O_2_) and body temperature maintained at 37 °C. A 350 μm silicon-coated monofilament (Doccol, CA, USA) was introduced in the external carotid artery and advanced through the internal carotid artery to occlude the MCA. After 60 min, the filament was retracted to allow reperfusion. Lidocaine (EMLA cream, AstraZeneca, Luton, UK) was applied locally to the wound and buprenorphine administered (Reckitt Benckiser Healthcare, Hull, UK, 0.025 mg/kg, s.c.) once, after reperfusion, to minimize pain. Sham animals received the same surgical procedure without occlusion. IL-1Ra (100 mg/kg, Biovitrum, Sweden) or vehicle (saline) were administered subcutaneously (s.c.) either acutely (at the time of occlusion) or in a delayed manner (24 h after occlusion). The experimenter was blinded to treatment until completion of all assessments and analysis. The dose and timing of administration of IL-1Ra was selected based on previous work [25]. Animals were randomly allocated to the following experimental groups: Sham + Veh (*n* = 6); Sham + IL-1Ra (*n* = 6); tMCAo + Veh (*n* = 10); tMCAo + IL-1Ra (*n* = 10; acute treatment, *n* = 5 and delayed treatment, *n* = 5). All sham animals were combined for analysis, as there was no significant difference between the subgroups groups. tMCAo animals treated with vehicle, either acute or delayed, did not differ significantly and were therefore combined for analysis. Gross neurological score was assessed daily as previously described [26]. Briefly, neurological deficits was scored by an observer blinded to the experimental conditions as follow: 0 = no observable deficit, 1 = flexion to the side contralateral to the ischemia whilst elevated, 2 = spontaneous circling to the contralateral side, 3 = falling to the contralateral side and 4 = no spontaneous movement.

### 2.4 Magnetic resonance imaging (MRI)

Brain infarct was evaluated early, 48 h after injury, by magnetic resonance imaging (MRI). Anesthetized animals (isoflurane: 4% induction, 1.5% maintenance) were scanned using a Magnex 7-Tesla horizontal-bore magnet (Agilent Technologies, UK) interfaced to a Bruker Advance III console (Bruker Biospin, UK) with a separate volume-transmit surface-receive radiofrequency head surface coil. *T*_2_-weighted images were acquired using a fast spin-echo pulse sequence (repetition time/effective echo time = 4800/60 ms, 8 echoes, field of view: 40 × 40 mm, matrix: 256 × 256, 2 averages, 25 slices of 1 mm). Infarct size and edema were determined using Image J (NIH Image, US) by an observer blinded to the experimental conditions.

### 2.5 Skilled reaching

Skilled forelimb reaching (SR) was assessed as previously described [27] with slight modifications. Training and testing sessions consisted of each rat reaching for 20 food pellets (40 mg, LBS Biotechnology, Surrey, UK). Training was considered completed once the animals reached a plateau of reaching success for at least 3 days. A successful reach was defined as the food pellet being grasped, retracted through the reaching box slit and brought to the mouth. An obtained pellet was defined as the animal being able to get and eat the pellet (with multiple attempts, possible drops, use of both paws or of the tongue by getting the pellet closer to the box opening). Once prior to surgery and on D6 and 25 post-surgery, the testing session was recorded with a videocamera and Any-Maze Tracking software (Stoelting Co, Dublin, Ireland) (30 frames/s). Qualitative analysis of limb movements was performed by frame-by-frame analysis of 3 successful reaches of each recorded testing session as previously described [27]. Briefly, 11 components of the reaching movements were assessed: (1) Orient, (2) Limb lift, (3) Digits close, (4) Aim, (5) Advance, (6) Digits open, (7) Pronation, (8) Grasp, (9) Supination 1, (10) Supination 2 and (11) Release, and 35 subcomponents were scored based on the following scale: 0: movement absent, 0.5: movement present but abnormal, 1: movement normal. Quantitative analysis of reaching included percentage of successful reaches and percentage of obtained pellets (which included successful reaches) on each testing day. All analysis was performed by an observer blinded to the experimental conditions.

### 2.6 Skilled walking

Skilled walking (SW) was assessed as described previously [28]. Briefly, animals were allowed to cross a one-meter long horizontal ladder with irregularly placed round metal rungs. This particular pattern was used to minimize the animal memorization of the task and instead be based solely on their motor capacities. The same pattern was maintained for both test sessions. Each test session consisted of the animal crossing the ladder three times without stopping (maximal number of five trials). Sessions were filmed to allow frame-by-frame analysis of foot placement on the rung using a 7-category rating system as previously described [28]. Foot placement score of 0–6 (0 = total miss, 1 = deep slip, 2 = slight slip, 3 = replacement of the limb, 4 = correction of limb position, 5 = partial placement, and 6 = correct placement) was given to each limb for each placement and scores averaged for each trial. The number of errors (defined as a score of 0, 1 or 2) in each trial was also assessed and averaged for the three trials. Since impairment after brain injury differed between forelimbs and hindlimbs, they were analyzed separately.

### 2.7 Open field and social interaction

On D7 and D26 after brain injury the animals were placed in the center of a 1-m square open field (Stoelting Europe, Ireland) and allowed to explore freely for 5 min while being filmed and tracked using the ANY-maze behavior tracking software (Stoelting Europe, Ireland). Total distance traveled and exploratory behavior was analyzed. The 5 min time interval was selected from preliminary analysis in order to allow time for the animal to explore and get familiarized with the environment. After 5 min, an unfamiliar animal (age and weight matched) was introduced to the arena (in the opposite quadrant) and both rats were allowed to freely interact for 5 min. Active social interactions (both number and length) made by the animal of interest was recorded and defined as sniffing, following or grooming the unfamiliar rat. The number of interactions, time spent interacting and the latency to first interaction was analyzed using the ANY-maze behavior tracking software (Stoelting Europe, Ireland) by an experimenter blinded to the experimental conditions.

### 2.8 Elevated plus maze

On D8 and 27 after surgery the anxiety levels of the animals was evaluated using the elevated plus maze (EPM) apparatus (Stoelting Europe, Ireland). Animals were placed in the center of the platform facing one of the open arms and allowed to explore the maze for 5 min while being recorded (ANY-maze behavior tracking software, Stoelting Europe, Ireland).

### 2.9 Forced swim test

On D9 and 28 despair behavior was assessed using the forced swim test (FST) by placing the animal in a cylinder (1 m deep and 45 cm diameter) containing water at room temperature for 5 min. Behavior was recorded and analyzed for immobility duration and latency to first float using ANY-maze behavior tracking software (Stoelting Europe, Ireland).

### 2.10 Tissue processing and histological analysis

On the last day (D28), animals were anesthetized (isoflurane 4%) and transcardially perfused with ice-cold 0.9% saline followed by 4% PFA. Brains were removed, post-fixed overnight at 4 °C in 4% PFA/20% sucrose, and transferred to 20% sucrose for 24 h. Thirty μm thick coronal sections were cut using a sledge microtome (Bright Instruments, Cambridge, UK) and stored in cryoprotectant solution (30% ethylene glycol, 20% glycerol in PBS) at −20 °C until used. For hematoxylin & eosin (H&E) staining, brains were mounted on Superfrost slides (Fisher Scientific, Loughbourough, UK) and allowed to dry overnight prior to staining as previously described. Residual infarct volume was measured on H&E stained sections taken throughout the brain (every 1 mm). Infarcted regions were manually delineated using Image J (NIH Image, US) by an investigator blinded to the experimental conditions. For immunohistochemistry (IHC) and immunofluorescence (IF), free-floating brain sections were processed as previously described [29]. Primary antibodies against myelin basic protein (MBP, 1:100, Millipore, Oxford, UK), ionized calcium binding adaptor molecule 1 (Iba1, 1:500, Wako Chemicals, VA, USA) or glial fibrillary acidic protein (GFAP, 1:500, Millipore, Oxford, UK) with the following secondary antibodies: anti-mouse-HRP, anti-rabbit Alexa-594, anti-mouse Alexa-488 (1:500, Life Sciences, Paisley, UK), were used. Sections were then mounted onto Superfrost slides and mounted with DPX (Fisher Scientific, Loughbourough, UK) or Prolong antifade medium containing Dapi (Life Sciences, Paisley, UK).

### 2.11 Statistical analysis

Data are presented as mean ± standard error of the mean (SEM). Kruskal–Wallis with Dunn's post-test or two-way ANOVA with Bonferroni's post-test were performed, as appropriate (see details in figure legends), using GraphPad Prism (Version 6.0, GraphPad Software, CA, US). Data were considered significant when *p* < 0.05.

## 3 Results

### 3.1 Acute anti-inflammatory treatment protects against brain damage and gross neurological dysfunction after cerebral ischemia

**Fig. 2:**
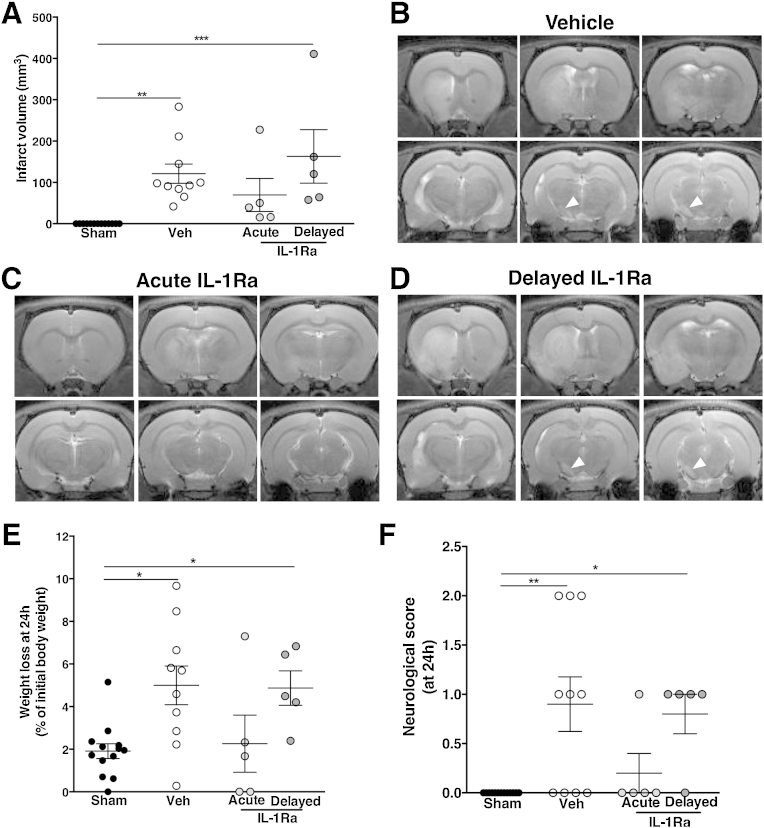
Early brain damage analysis and neurological score assessment. At 48 h after injury both vehicle and delayed-IL-1Ra treated animals showed similar infarct volume (A). Damage was mainly localized to the striatum and remote injury was seen in the substantia nigra, as seen on representative images from *T*_2_-weighted MRI (white arrow, B). Acute administration of IL-1Ra decreased infarct volume and protected against remote damage (C) although delayed administration of IL-1Ra showed both striatal and substantia nigra damage as did vehicle treated (D). Weight loss at 24 h after injury (E) and neurological score assessment (F) showed that vehicle-treated and delayed-IL-1Ra treated animals performed worse. Data presented as mean ± SEM. *p < 0.05, **p < 0.01, ***p < 0.001 using Kruskal–Wallis with Dunn's post-test (*n* = 5–10).

Exposure of animals to 60 min tMCAo led to brain injury, as detected by MRI performed 48 h after the insult (Fig. 2A). Infarct was consistently observed within the striatum and extended to the cortex/hippocampus (70% of the cases) as well as the remote ipsilateral substantia nigra (SN), where damage was observed in 90% of the cases (Fig. 2B). Animals treated with IL-1Ra acutely (at the time of occlusion) had 43% smaller infarcts but delayed-IL-1Ra treated animals (treated at 24 h post-injury) had similar infarcts to the vehicle-treated group (Fig. 2A–D). In 90% and 100% of the tMCAo animals treated with vehicle or delayed IL-1Ra, respectively, remote damage was observed in the SN (white arrowhead in Fig. 2B and D), while this was observed in only 20% of tMCAo animals treated acutely with IL-1Ra. Edema was only observed in animals treated with vehicle (41 ± 15 mm^3^, *p* < 0.01) or delayed IL-1Ra (77 ± 33 mm^3^, *p* < 0.001) (data not shown). Initial weight loss (24 h after injury) was observed in tMCAo animals treated with vehicle or delayed IL-1Ra only (Fig. 2E). Based on the neurological score at 24 h, both vehicle-treated and delayed IL-1Ra groups performed significantly worse than sham animals (Fig. 2F). However, from 48 h and onward, there was no difference in gross neurological scores between any of the experimental groups, as all went back to levels of sham animals (data not shown). This reflects the high capacity of the animals to rapidly improve in most of their reflexes and basic neurological capacities after brain injury and the need for more detailed analysis of function.

### 3.2 Acute IL-1Ra treatment promotes functional recovery whilst compensation is observed in vehicle-treated animals through fine motor assessment

**Fig. 3:**
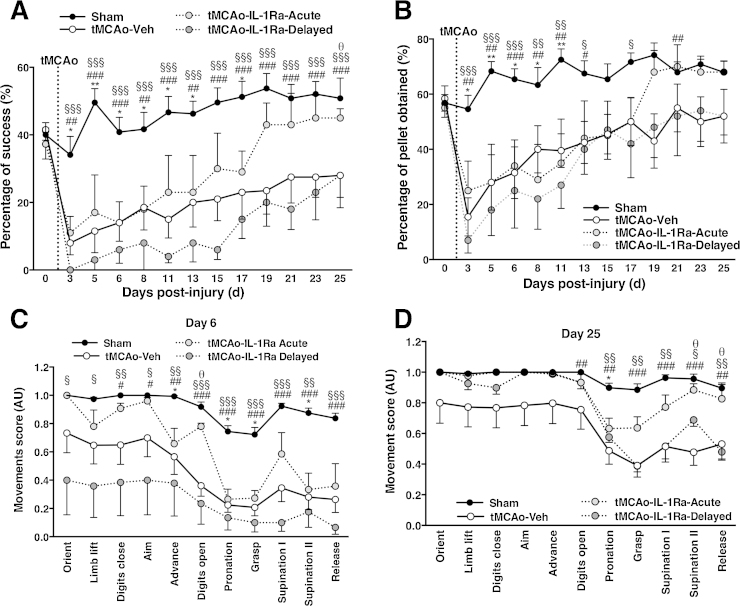
Skilled reaching assessment after brain injury. Animals in all three treatments groups showed evidence of impairments early after injury (D3) as seen by the decreased success rate and displayed some degree of recovery by D25 although vehicle and delayed IL-1Ra treated animals still showed significant impairments (A). Even if animals were impaired in their reaching success, they were able to obtained pellets (B) with the use of compensatory mechanisms. Detailed analysis of successful reaches at D6 post-injury allowed to further dissociate between groups as all had similar success rate, but several components of movements were protected by acute IL-1Ra treatment (C). By D25, detailed movements analysis showed impairments, especially in pronation, grasp and supination (D). Data presented as mean ± SEM. *p < 0.05, **p < 0.01 acute IL-1Ra vs. sham; ^#^*p* < 0.05, ^##^*p* < 0.01, ^###^*p* < 0.001 vehicle-treated vs. sham; ^§^*p* < 0.05, ^§§^*p* < 0.01, ^§§§^*p* < 0.001 delayed IL-1Ra vs. sham; ^θ^*p* < 0.05 vehicle-treated vs. acute IL-1Ra-treated, using two-way ANOVA with Bonferonni's post-test for A and B and Kruskal–Wallis with Dunn's post-test for each movement components in C and D (*n* = 5–10).

Fine motor function was first assessed using the skilled reaching task. Prior to surgery, all animals presented with the same level of performance in the task (Fig. 3A, D0). Animals were then tested every other day from D3 post-injury until D25 and filmed twice (D6 and 25) to allow detailed movement analysis. On D3 after brain injury the animals from all three experimental conditions presented significant impairments characterized by decreased success rate in the task (Fig. 3A). All experimental groups significantly improved between D3 and D25 (Fig. 3A). However, only the animals treated acutely with IL-1Ra returned to the same level as sham animals, showing a 50% success rate at D25, and performed significantly better than vehicle-treated animals (*p* < 0.05, Fig. 3A). In contrast, both vehicle-treated and delayed IL-1Ra groups showed significant impairments at D25 (both *p* < 0.001 vs. sham). Although impairments were still detectable at D25, animals were able to obtain the pellets, even if not in a successful manner (percentage of obtained pellets includes successful reach). The difference between the impairment observed in the success rate at D25 (Fig. 3A) compared to the animals capacity to obtained pellets (Fig. 3B), is an indication of the development of compensatory mechanisms in order to achieve the task, even if not in a successful manner. Examples of compensatory mechanisms included pellet drop within the cage and grasp directly with the mouth, help from the contralateral paw and use of the tongue after getting the pellet closer.

Detailed qualitative analysis of the different movement components was performed early after injury (D6) when the experimental groups all presented similar degrees of impairments and at a later time point after injury (D25). At D6, even if all experimental groups globally showed a similar degree of impairment, detailed analysis of successful reach revealed that the animals capacity to orient, limb lift, close and open digits and aim were preserved in the group treated acutely with IL-1Ra while being impaired in those treated with vehicle or delayed IL-1Ra (Fig. 3C). The movement components pronation, grasp, supination and release were the most affected by injury in all groups (Fig. 3C). By D25, although animals treated acutely with IL-1Ra returned to control levels in term of success rate, detailed analysis of their movements showed sustained impairment in their pronation. Animals treated with vehicle or delayed-IL-1Ra both improved by D25 as compared to D6 but still displayed significant impairments in their pronation, grasp, supination and capacity to release the pellet (Fig. 3D).

### 3.3 Recovery of motor function assessed using the skilled walking task.

**Fig. 4:**
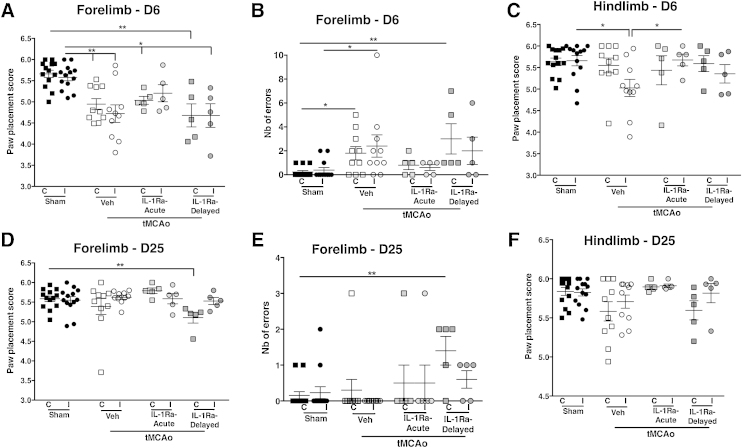
Skilled walking assessment using the horizontal ladder task. Impairments in the walking task were observed in both contralateral and ipsilateral forelimb of vehicle and delayed-IL-1Ra treated animals but only on the contralateral forelimb of acutely-treated animals (A). Increased number of errors was seen in both vehicle and delayed-IL-1Ra treated groups (B). Hindlimb function was only mildly impaired at D6 post-injury (D6) (C). By D25 impairments was observed in forelimb function only in the contralateral side of delayed-IL-1Ra treated groups (D) and in the number of errors (E). Hindlimb function had returned to sham level by D25 (F). Data presented as mean ± SEM. * = *p* < 0.05, ** = *p* < 0.01, using Kruskal–Wallis with Dunn's post-test (*n* = 5–10).

Skilled walking (SW) was also used to assess other aspects of fine motor skills, using the ladder test performed early (D6) and late (D25) after injury. Globally, there was no difference in the time needed for the animals to cross the ladder between any of the experimental groups. At D6, significant impairments were observed across experimental groups in forelimb usage, as seen by the decreased paw placement score (Fig. 4A). The number of errors (defined as a missed or nearly missed step) was significantly increased only in vehicle-treated animals (both contralateral and ipsilateral, *p* < 0.05) and delayed-IL-1Ra treated animals (contralateral only, *p* < 0.01) (Fig. 4B). Hindlimb use was mainly preserved and the only significant impairment observed was in the vehicle-treated group (ipsilateral, *p* < 0.05 vs. sham and *p* < 0.05 vs. acute IL-1Ra-treated) (Fig. 4C). By D25, animals in all groups improved with the only residual impairment observed in the contralateral forelimb of animals treated with delayed IL-1Ra (Fig. 4D and E). No significant impairment was seen in hindlimb usage at this time between any of the experimental conditions (Fig. 4F).

### 3.4 Skilled reaching success rate correlates with both early and late infarct size

**Fig. 5:**
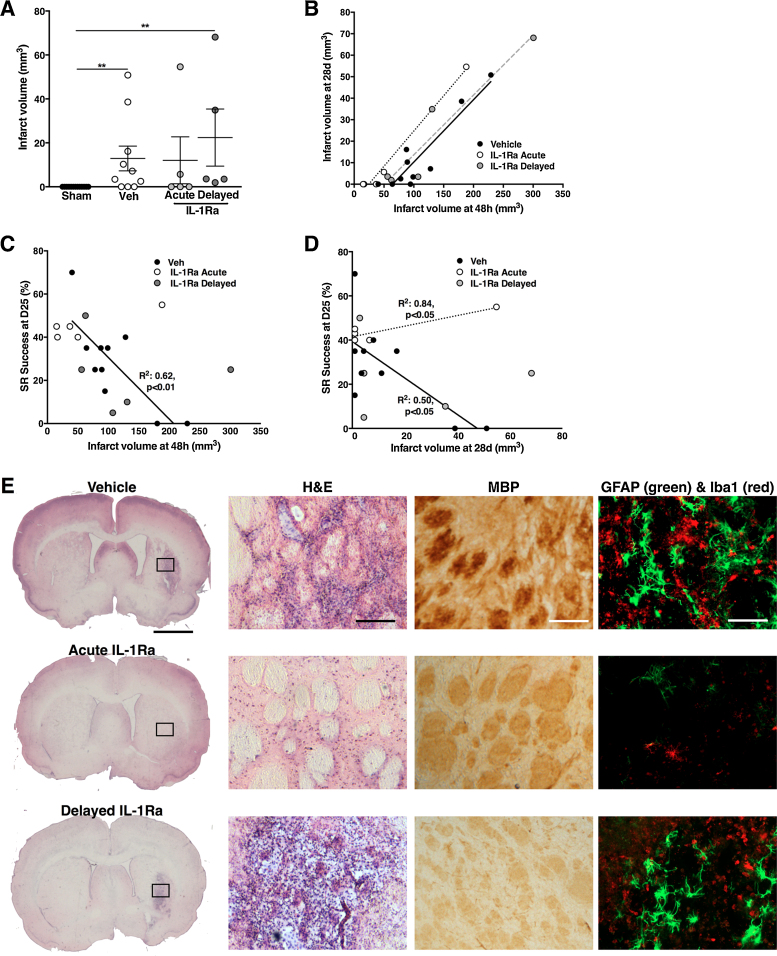
Infarct analysis at D28 and correlation with motor function. Infarct size at 28d (A) was strongly correlated with infarct volume detected at 48 h in all experimental groups (B). Infarct volume at both 48 h (C) and 28d (D) correlated with fine motor skills using the skilled reaching task in vehicle-treated animals only. Histological analysis of the infarct at 28d revealed increased cell number characteristic of glial scar within the striatum (H&E staining) with loss of architecture of the myelin fiber tract (MBP staining) and increased numbers of astrocytes and macrophages/microglia (GFAP and Iba1 staining) (E). Pearson correlation analysis in A–C. Representative images of histological analysis (E). Scale bar: 200 μm for whole brain and 100 μm.

Residual infarct was determined D28 after injury and was much smaller in all of the experimental groups as compared to that observed acutely (48 h) (vehicle-treated: 13 ± 6 mm^3^, *p* < 0.01 vs. sham; acute IL-1Ra: 12 ± 11 mm^3^, ns vs. sham; and delayed IL-1Ra: 22 ± 13 mm^3^, *p* < 0.01 vs. sham). There was a strong positive correlation between the initial infarct volume and the residual damage observed at D28 across all treatment groups (vehicle-treated: *R*^2^ = 0.85, *p* = 0.0002; acute IL-1Ra: *R*^2^ = 0.98, *p* = 0.001; delayed IL-1Ra: *R*^2^ = 0.91, *p* = 0.013) (Fig. 5A).

Infarct volume, analyzed acutely at 48 h, was negatively correlated with skilled reaching success rate at D25 after injury but this correlation was seen only in the vehicle-treated group (*R*^2^: 0.62, *p* = 0.007) (Fig. 5B). Residual infarct at D28 was weakly but significantly correlated with skilled reaching success rate at D25 in vehicle treated animals (*R*^2^: 0.5, *p* = 0.02). By contrast, there was a slight positive correlation in animals acutely treated with IL-1Ra (*R*^2^: 0.84, *p* = 0.03) without any correlation in the delayed-IL-1Ra treated group (Fig. 5C). Residual damage was localized mainly to the striatum and, in some cases, affected the motor cortex and amygdala. The damage was characterized by high cellular density, typically associated with a glial scar (Fig. 5D). Immunohistochemistry for myelin basic protein (MBP), showed a loss of the architecture of myelin tracts within the striatum, visible only in vehicle-treated and delayed IL-1Ra treated animals. Increased number of GFAP+ astrocytes with hypertrophied bodies and increased density of activated Iba1+ microglia/macrophages was also observed in the vehicle and delayed-IL-1Ra treated groups only (Fig. 5D). Neuroprotection through acute treatment with IL-1Ra was still visible at D28 as seen by the preserved myelin tracts and absence of glial scar (Fig. 5D). There was no difference in the histological analysis between acute IL-1Ra and sham animals (data not shown).

### 3.5 Decreased social interaction and depressive behavior after brain injury

**Fig. 6:**
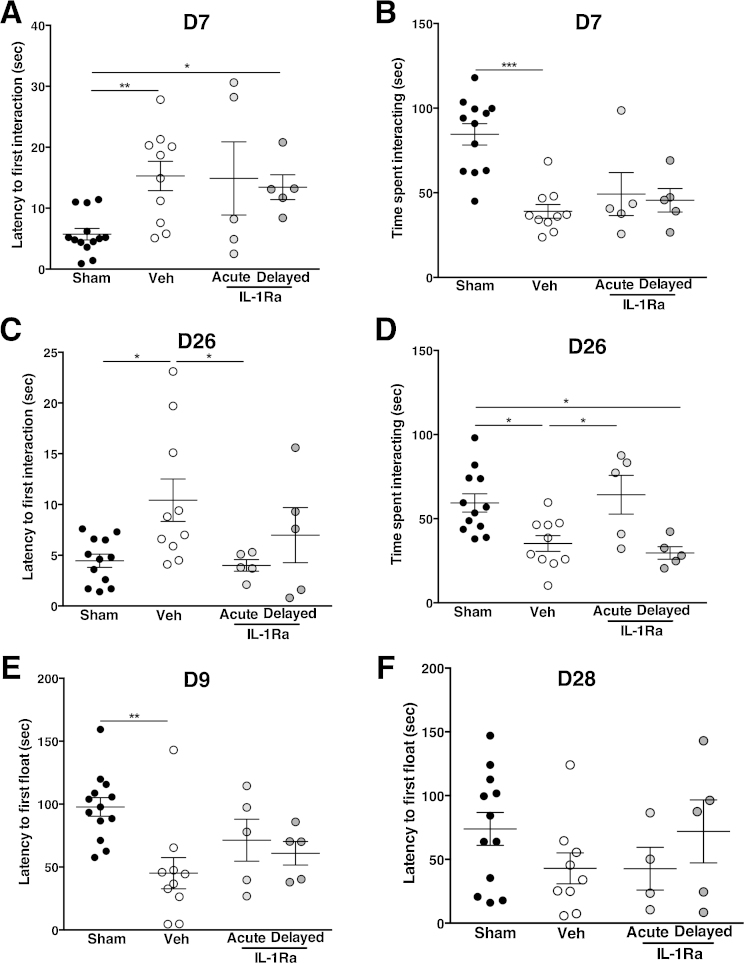
Sociability impairments and depressive behavior after brain injury. Analysis of sociability showed impairments in the latency to first interaction (A) and total time spent interacting (B) early after stroke (D7). These effects were sustained and still visible at D26 (C–D). Depressive behavior was analyzed using the forced swim test (FST) and vehicle-treated animals showed decreased latency to first float early D9 after injury (E) but by D28 (F) no differences was observed between groups. Data presented as mean ± SEM. *p < 0.05, **p < 0.01, using Kruskal–Wallis with Dunn's post-test (*n* = 5–10).

Although fine motor function was affected, and impairments clearly visible even at a late stage after injury, there was no difference observed in term of locomotion and gross motor skills (assessed using the open field test), with the animal displaying similar behavior in terms of their distance traveled and exploration pattern (data not shown). Social interaction, depression-like and anxiety-like behaviors were assessed at both early (D7–9) and late (D26–28) time points after injury. At D7 after injury, vehicle or delayed IL-1Ra-treated animals displayed a longer time to first interaction when placed with an unfamiliar, age and sex matched, animal and vehicle-treated animals spent less time interacting compared to sham animals (Fig. 6A and B). Although animals treated with IL-1Ra (both acute and delayed) showed a trend to a decreased time spent interacting at D7 this was not significant (Fig. 6B). By D26, animals treated acutely with IL-1Ra were protected against sociability defect but vehicle treated animals still showed significant impairments in their sociability, as seen by the longer time to first interaction (*p* < 0.05 vs. sham and acute IL-1Ra-treated) as well as decreased total time spent interacting (*p* < 0.05 vs. sham and acute IL-1Ra-treated) (Fig. 6C and D).

Depressive-like behavior was observed in vehicle treated animals, as seen by the shorter latency to first float as compared to sham animals, in the FST test of despair/depression (Fig. 6E). Animals treated with IL-1Ra, irrespective of the timing of administration, were protected and did not present depressive-like behavior (Fig. 6E). By D28 there were no differences between any of the experimental conditions (Fig. 6F). Anxiety-like behavior, determined using the EPM test, was similar across all experimental groups (data not shown). Unlike fine motor function, social interaction and depression-like behavior were not correlated with infarct size (data not shown).

## 4 Discussion

Translation of new neuroprotective treatments from the preclinical to the clinical setting in stroke has been a challenge. The use of more detailed analysis of functional outcomes after brain injury might offer possibilities to better correlate results obtained from animal models with the clinical situation, and better assess the therapeutic potential of new treatment options. In the present study we used fine motor tasks as well as emotional assessments in order to assess long-term impairments after stroke and the effect of IL-1Ra. The skilled reaching task was especially sensitive in the assessment of impairments, even in animals with small infarct, and allowed differentiation between functional recovery and compensation. Defects in sociability and depressive-like behavior were also observed after injury. Acute IL-1Ra administration, improved long-term functional recovery, as opposed to compensation in animals treated in a delayed manner without any effects on infarct volume. These observations indicate enhanced efficacy of early neuroprotection compared to late treatment, with sustained functional protection.

The skilled reaching task showed high sensitivity to fine impairments even 1 month after injury. This is in agreement with previous studies showing that functional improvement after discrete cortical ischemia induced by photothrombosis or endothelin-1 injections mainly relies on the adoption of behavioral compensatory mechanisms and detailed analysis of skilled reaching as proven useful to detect these impairments [13,30]. In various follow-up studies, genuine recovery, i.e., restoration of the original movement patterns in skilled reaching task after an injury has rarely been observed [31]. Even if the underlying mechanisms leading to compensation may be different between these models based on the lesion location and extent, the skilled reaching task is highly sensitive to slight changes in movement and likely involves activity-dependent recruitment of intact neuronal circuits [32]. The differentiation between recovery and compensation is also of high importance in order to assess the route of action and rehabilitation potential of new therapeutic approaches. Contrary to previous reports [7–9,14], we found a correlation between infarct volume, measured either early or late after injury, and success rate in the skilled reaching task although there was no correlation found with the other tests. In a recent study, Balkaya et al. [7] used a wide range of motor tests in order to address long-term outcomes after tMCAo in mice and did not find any correlation between infarct and outcomes. Most of the tests used in this study did not show differences between groups after 1–2 weeks post-lesion but a few, mainly assessing laterality, did show long-lasting impairments [7]. These differences might be due to the model used (mice vs. rat in the present study) but clearly highlights the importance of combined use of multiple tests with the skilled reaching task as the information obtained with the latter cannot be acquired any other way and is uniquely sensitive to different types of brain injury. Furthermore, other studies have shown a correlation between histological assessment and functional outcomes [11]. Combined use of multiple tests evaluating different aspect of behavior, alongside longitudinal assessment of brain injury allows one to gather a global understanding of long-term outcomes, with imaging techniques allowing detailed white matter tract analysis increasingly being used [33].

Acute neuroprotection remains challenging in the clinic as there are practical delays between presentation and treatment administration. In accordance with the present study, previous reports showed the protective effect of subcutaneous administration of IL-1Ra in models of cerebral ischemia but all early after injury (24 h) [26,34,35]. Furthermore, Pradillo et al., did not detect significant changes in behavioral [34], which emphasize the necessity to use more sensitive functional assessment. Here we evaluated the long-term effects on fine motor function after cerebral ischemia as well as the long-term impact of IL-1Ra treatment. Assessment of fine motor function revealed that a decrease in initial infarct volume through acute IL-1Ra administration led to enhanced recovery of the original movement ability, whilst animals with delayed IL-1Ra treatment showed evidence of compensation, similarly to the vehicle-treated group. It is important to note that acute IL-1Ra treatment led to protection and recovery which was sustained and still observed after 1 month, as acute neuroprotective properties are not always associated with sustained protection [10]. Since infarct measurement at D28 was positively correlated with SR success rate (at D25), only in animals treated acutely with IL-1Ra, we can speculate that IL-1Ra not only promotes functional restoration through neuroprotection but likely through effects on plasticity as well, although the underlying mechanisms requires further investigation. This is in line with the known detrimental actions of pro-inflammatory cytokines on damage progression and repair mechanisms after brain injury [32]. Astrogliosis and microgliosis were not observed in animals treated acutely with IL-1Ra, but still detected in animals treated in a delayed manner, this would suggest that early neuroprotection is key in order to minimize the long-term effects of brain injury as compared to a delayed anti-inflammatory intervention. A shorter delay in IL-1Ra treatment after cerebral ischemia (up to 3 h) was shown to be neuroprotective [36] and we could expect a similar long-term protection in motor function with administration within that time window.

Since emotional disturbances and motor function are mediated by distinct neural pathways, it is important to evaluate both after brain injury, especially since defect in sociability and depression can have a negative impact on functional recovery. Pro-inflammatory cytokines are also potent inducers of these emotional changes and have been shown to induce depressive-like behaviors in animals [23,37]. It is important to address these behaviors (namely depression, sociability, etc.) in any pathological setting with an inflammatory component as they are likely contributors to the outcomes and anti-inflammatory treatment could potentially be used to decrease the impact of cytokines on brain function. In the present study we did not observe protection of these behaviors independently of motor function as both were preserved in animals treated acutely with IL-1Ra, but not with delayed treatment. However, IL-1Ra was previously reported to be beneficial in post-stroke depression when administered with a 5d delay, therefore not protecting against brain infarct [38] although motor function was not assessed. More studies are needed in order to differentiate between motor function and sociability and depression after cerebral ischemia in order to determine their potential role in recovery. Also the precise timing and mode of administration of delayed IL-1Ra still needs further investigation. Nevertheless, the response to acute IL-1Ra represents an outstanding example of genuine restoration of the original motor function based on neuroprotective actions.

In summary, using a comprehensive behavioral test strategy, the present results highlight that skilled movements bear predictive value for symptomatic outcomes after ischemic brain injury with particular sensitivity to therapeutic success and associated neuronal protection and plasticity. Furthermore, early neuroprotection with IL-1Ra enhanced functional recovery and protected against sociability defect and depression, which further emphasizes the importance of targeting the initial infarct and extending the therapeutic window in order to promote recovery.

## Conflict of interest

NJR is a non-executive director of AstraZeneca but this has no relationship to the research described here. The other authors declare no competing financial interests.
